# The Association Between Spousal Support And Attitude For Gerontechnology Via Facilitating Conditions

**DOI:** 10.1093/geroni/igab046.3348

**Published:** 2021-12-17

**Authors:** Susanna Joo, Seong Hee Kim, Changmin Lee, Kwang Joon Kim, DaeEun Kim, YoonMyung Kim, Hey Jung Jun

**Affiliations:** 1 Yonsei University, Seodaemun-gu, Seoul-t'ukpyolsi, Republic of Korea; 2 Sungkyunkwan University, Seoul, Republic of Korea; 3 Yonsei University, Seoul, Seoul-t'ukpyolsi, Republic of Korea; 4 Severance Hospital, Yonsei University College of Medicine, Seoul, Seoul-t'ukpyolsi, Republic of Korea

## Abstract

This study examined the mediating effects of facilitating conditions (FC) on the association between types of social support providers and attitude toward using gerontechnology (AUG) or between types of social support and AUG. The sample was 256 older Koreans having a partner and children (N=256; 66-88 years old; M=69.91; SD=4.19). The dependent variable was AUG in terms of an exoskeleton robot for exercise. There were two kinds of independent variables: 1) four types of social support provider (spouse, children, siblings/relatives, and friends/neighbors), and 2) four types of social support (emotional, instrumental, physical, and financial support). Mediating variable was calculated as the mean of FC from five questions. There were two analytic steps: 1) structural equation modeling with four latent variables about types of social support provider, and 2) path analysis with four types of social support if only for significant providers at the first analysis. The results from the first step of analysis showed that only social support from spouses had a significant effect on AUG via FC to use gerontechnology. In the second step of analysis, emotional support from spouses was associated with the higher level of AUG via FC to use gerontechnology. The findings could shed light on the salience of emotional support from spouses in terms of the positive attitude on technology usage in later life through enhancing FC for technology acceptance.

